# The empowerment of elderly patients with chronic obstructive pulmonary disease: Managing life with the disease

**DOI:** 10.1371/journal.pone.0174028

**Published:** 2017-04-03

**Authors:** Zahra Fotokian, Farahnaz Mohammadi Shahboulaghi, Masoud Fallahi-Khoshknab, Ali Pourhabib

**Affiliations:** 1 Department of Nursing, Ramsar Nursing Care Research Center, School of Nursing and Midwifery, Babol University of Medical Sciences, Babol, Mazandaran, Iran; 2 Social Determinants of Health Research Center, University of Social Welfare and Rehabilitation Sciences (USWR), Tehran, Iran; 3 Department of Nursing, University of Social Welfare and Rehabilitation Sciences (USWR), Tehran, Iran; 4 Department of Nursing, School of Nursing and Midwifery, Babol University of Medical Sciences, Babol, Mazandaran, Iran; Universite de Bretagne Occidentale, FRANCE

## Abstract

Chronic obstructive pulmonary disease (COPD) is a serious health problem that has significant effects on the life status of elderly persons. Use of the empowerment approach is necessary for health promotion in older people with COPD, but little attention has so far been paid to all the dimensions of empowerment in the management of COPD, which would provide useful knowledge regarding elders with COPD. This article reports on a study exploring people’s experiences of the empowerment of older people with COPD. This study adopted an exploratory qualitative design and was carried out using grounded theory methodology. Grounded theory was considered appropriate for this study because of its focus on how people respond to and act on the problems that they encounter. We collected data by conducting in-depth semi-structured interviews and taking field notes. Twenty-four participants were selected through purposive sampling.

The results showed that in encountering the complexity of disease and in response to difficulties induced by COPD, three strategies were applied. Elderly persons with COPD, their family caregivers, and professional team members engaged in “managing life with COPD,” “striving to keep abreast of life,” “preparing for battle with disease,” and “helping to stabilize the elder’s life.” The outcome of these strategies was “co-existence with disease.” The potential of “managing life with COPD” was influenced by the following factors: “co-existence with ageing,” “personal potential,” “a challenged health system,” and “weak social support.” “Managing life with COPD” enables the elder to feel in control and live optimally. This is a fragile balance, however, and the unpredictability of COPD can tip the elder into “self-efficacy.” Understanding the experiences of the empowerment process of older people with COPD can help health professionals provide more focused elderly care.

## Introduction

Chronic obstructive pulmonary disease (COPD) is becoming an increasingly prevalent health problem globally, accounting for massive healthcare expenditure [1]. By 2020, this disease is expected to cause 7% of all deaths worldwide (4–5 million people annually). The prevalence of COPD increases with age, with a five-fold increased risk reported for those aged over 65 years, compared with patients aged less than 40 years [2]. The condition affects the well-being of an affected older person and the degree to which this subgroup can be ‘physically active’ and participate in social relations [3, 4]. Predominant symptoms include fatigue-induced hypoxia and restrictions in daily living activities [5]. These limitations increase physical disability and elders’ dependence on other people [6]. Although COPD results in problems in elders’ ability to maintain control over the disease and their lives, studies have shown that empowerment programs constitute recommended non-pharmacological treatment for COPD, with considerable evidence of benefits to older patients. This demonstrates that empowerment programs are central to reducing the severity or frequency of exacerbations, preventing hospitalization, and improving health-related quality of life [7].

COPD is a serious health problem that has significant effects on healthcare services and the life status of older persons. Use of the empowerment approach is necessary for health promotion and for enabling the efficacy required by elderly persons with COPD to have control over the disease. However, previous results have revealed that many older people were unaware of strategies that can be used to manage COPD. This is supported by Hyland (2005), who found that patients with COPD need to be involved in the development of patient information resources. The results of Robinson’s (2010) study showed that 30% of elderly patients with COPD are not aware of management practices and control measures over their condition, and not aware of control measures over their condition’, and identified feelings of powerlessness and poor disease management [8]. This points to a need to empower older people with COPD, to enable better self-management and behavioral changes aimed at improving efficacy and quality of life, and reducing disability and healthcare costs [9]. Empowerment will enable elderly patients to actively participate in their own treatment and plan, access vital information, and make decisions [10].

In spite of the importance and use of the empowerment concept, little attention has so far been paid to all the dimensions of empowerment in the management of diseases such as COPD. This study aimed to explore the process of empowerment among older persons with COPD. Despite the importance and application of the concept of empowerment, lack of sufficient knowledge all aspects in the management of chronic diseases, particularly COPD. In the majority of studies in the field of chronic disease, implementation of an educational program was considered equal to empowerment. These interventions, in which quantitative methods were employed, were used to impart elderly patient education [11]. However, quantitative studies cannot capture the essence of the empowerment concept. While patient education is one aspect of empowerment process, and one of the tasks of the professional team members in empowerment process is education. More studies in relation to empowerment interventions have been conducted as elderly patient education by using a quantitative method in social and cultural context of old people with chronic disease [10].

There is an abundance of vague definitions of the empowerment concept by nurses and healthcare providers; this shows that knowledge regarding the empowerment of Iranian e**lders** with COPD is not well developed. Considering the consequences of empowerment (i.e., self-efficacy, well-being, and QOL promotion) for older people with COPD, it is important to understand the meaning of COPD, particularly to elderly Iranian persons with COPD, as well as how they respond to the disease. The aim of this study was to illuminate the experiences of empowerment among elderly patients with COPD, their family caregivers, and healthcare providers in the Iranian context. This can be understood by studying the interactions of older people with their families, healthcare providers, and society.

## Methods

This study adopted an exploratory qualitative design and was carried out using Grounded theory methodology, which is mainly based on Corbin and Strauss’s paradigmatic model; its focus is on how people respond to and act towards problems that they encounter. Goal of a grounded theory study is to discover the participants' main concern and how they continually try to resolve it. The questions the researcher repeatedly asks in grounded theory are "What's going on?" and "What is the main problem of the participants, and how are they trying to solve it?" These questions will be answered by the core variable and its sub-cores and properties in due course [12]. Empowerment concept is an extensive concept [10]. Prevalent definitions of empowerment are therefore mainly based on the view that autonomy is recognized in people’s self-determination, and that empowerment is a procedural process of giving (provider to patient) and taking (patient from provider) power. Patient empowerment needs to be seen as a dynamic and creative process that is shaped by the individual’s own activity and yet acknowledges the individual’s dependence on others [13]. This concept is an interactional concept, and in the process of empowerment, the elder with COPD, family and professional team members respond to and act towards problems that they encounter [13]. Therefore the exploring of empowerment process with grounded theory (it explores the process of empowerment) in the elders with COPD can help to identify factors facilitating empowerment and the model of employing the process in the elderly with COPD. Then the exploring the existing process via grounded theory can help to Healthcare providers, and health policymakers in obstacles removal and reinforcement facilitators of the process of empowerment.

We collected data by conducting in-depth semi-structured interviews and making field notes. Criterion-based and purposive sampling was employed [12]. The following four criteria were used to select participants: (i) Persons with COPD aged 60 years or older (in Iran, >60 years is considered old age); (ii) Persons with COPD have an interest in and the ability to explain own experiences, (iii) have a minimum of 5 years’ experience in caring for patients with COPD (for healthcare providers), (iv) be a family caregiver for elderly relatives with COPD (for family caregivers). Study participants (n = 24) were recruited from an academic hospital under the management of the National Research Institute of Tuberculosis and Lung Diseases (NRITLD) in Tehran and two district hospitals in the north of Iran. Considering that empowerment is an interactional phenomenon [12], interviews were conducted with older people with chronic diseases and with their family caregivers and healthcare providers. We collected data through in-depth semi-structured interviews and field notes. Twenty-four participants were selected using purposive sampling. Purposive sampling was superseded by theoretical sampling once data analysis commenced [12]. Therefore, 24 in-depth interviews were conducted with 15 elderly persons with COPD, 5 healthcare providers, and 4 family caregivers (Table 1). We tried to include participants with different experiences of empowerment, based on age, sex, marital status, education, socio-economic status, employment status, and duration and severity of disease, to ensure theoretical saturation. The Abbreviated Mental Test, which yields a score of 6 or more, was used to screen people for cognitive impairment. The main participants (elders with COPD) could invite someone to support them while attending the interview and could stop the interview if they became breathless or did not want to continue. Data were collected from March 2012 to February 2014. The interview venues were chosen by the participants, for their convenience. In the case of interviews performed by the first author, the participants were interviewed at their homes (n = 8), the hospital (n = 8), the rehabilitation clinic (n = 4), or their workplace (n = 4). The interviews lasted 30–100 minutes, based on the participants’ tolerance and interest. The initial questions were broad, to encourage participants to speak freely and recount their personal experiences in relation to the objective of the study. Examples of the questions asked are, “What would empower you?” and “What are your experiences of empowerment?” Then, the participants’ answers during the interviews were coded and analyzed, followed by more follow-up questions to the participants. Data collection and analysis occurred concurrently. Data were analyzed based on Corbin and Strauss’s (2008) approach. For data analysis, we also used analytical tools such as asking questions, constant comparison, theoretical comparison, and considering the different meanings of a given word. The constant comparative technique was used to analyze the data. Analysis began with the repeated reading of the transcript, to facilitate immersion in the data. Open codes were initially attached to experiences, actions, thoughts, feelings, and events related to empowerment, as experienced by elders with COPD. After extracting the codes and subcategories, the main categories (or themes) were extracted. Following the generation of concepts, synthesis of the structures, and determining their relationships, the relevant theory was generated and explained. In this study, we carefully selected cases and conducted triangulation and external checks to ensure research rigor. Linking open codes resulted in the emergence of tentative categories. Coding became progressively more conceptual, as analysis progressed. Memos and diagrams were used to help reveal the relationships between categories. This facilitated the emergence of the core category.

**Table 1:** Strategies applied for "Managing life with COPD".

Participants	The elders with COPD(n = 15)	Family caregivers (n = 4)	Healthcare providers(n = 5)
Main Categories	Striving to keep abreast of life	Help to stabilize the elder’s life	Preparing for battle with disease
**Subcategories with** **related quotations**	**1.Information seeking** *I asked about what I can eat; should I follow a diet for my disease*? *The doctor told me ‘no*, *you can eat everything*, *you do not need any diet*. (Participant 5, a 77-year-old woman) **2.Participation in care** *If they feel breathless*, *they could use the devices for initial treatment at home*. *… When experiencing breathlessness*, *I use oxygen*, *spray myself*. (Participant 7, an 80-year-old woman) **3.Independence seeking** *I do all my own work*. *I do not need other people and I am not dependent*. *I am not bedridden and do not need my wife or kids to do things for me because of my disease*. (Participant 1, a 70-year-old man) **4.Promotion of psychosocial capacities** *That is right; the disease has been bothering her for months*, *but I will do my best to make her well*, *to live with her happily like in the past*. *Well*, *we used to go to the park every morning before November; we did it to boost my mom’s morale*. (Participant 6, a family caregiver) **5.Learning the life with COPD** *I live with shortness of breath nearly 11 years old*. *I know what make me bad opponent*. *When I get up*, *I use my drugs immediately*. *A lot of times I'm good; but if my opponent*, *I'll hospital*. *(Participant 3*, *a 86-year-old man)*	**1.Cooperation with professional team members** *Elders who have good family support get better answers than those who live alone; not a hundred percent*, *but it is less likely*. *In addition*, *an elderly who may not see or hear well does not have a good relationship with the outside world*. (Participant 16, pulmonologist) **2.Support for the elderly** *I also seek to ensure the welfare by providing physical*, *emotional*, *and financial support for my mother*. (Participant 6, a family caregiver) **3.Improve patient care skills** *When I saw my mother’s disease progress day by day*, *I followed up with the doctor*. *I know a lot about my mother’s disease*, *about drugs*, *respiratory aid devices*. *As the saying goes*, *I am an ‘expert*.(Participant 12, a family caregiver)	**1.Using effective training techniques** *Initially*, *during the first 15–20 minutes*, *we only check what they learned the previous time or whether they learned anything or no*, *and also examine if they perform activities correctly*. *Then*, *we ask questions to see how much they are learning*. *After ascertaining about their learning*, *we decide what to do for their physical therapy*, *because of their conscious fluctuations*, *one day they are sensible and the other day they are confused; this is why we have to teach their families*, *too*. (Participant 18, a physiotherapist) **2.Accepting the role of the elder-family in treatment** *We are providing training on tobacco cessation*, *no exposure to very cold or hot environments*, *no disposal of stimulants*, *[and] drug use*, *and they cooperate with us*. *We talk about tobacco cessation*, *no exposure to very cold or hot environments*, *no disposal of stimulants*, *drug use*, *etc*. *They are cooperating with us*. (Participant 16, pulmonologist) **3.Development of knowledge and professional skills** *There are two physiotherapists in the hospital*, *but they are responsible for the ‘whole hospital*, *like’ or ‘there is staff shortage'*, *but they try to work with all patients*. *(Participant 15*, *head nurse)*

Steps were taken to ensure the credibility, auditability, and fittingness of this study. Methodological rigor was enhanced by the rigorous application of the grounded theory methodology and techniques. The consistency of coding was monitored throughout the analysis. The researchers discussed, questioned, and agreed on emerging category labels and definitions at all stages of analysis. To evaluate consistency, we individually coded each interview. To strengthen the credibility of the study, the findings were presented to experts in the field, including elders with COPD, their family caregivers, and professional team members, all of whom agreed that the theory was both “recognizable” and “fit” with their experiences [12].

The study was approved by the Research Ethics Committee at the Welfare and Rehabilitation Sciences University in Iran (Ethical code: USWR.REC.1393.231). The participants were informed about the study verbally and were assured of confidentiality and anonymity. They were informed that they could withdraw from the study at any time. The participants provided written informed consent before the interviews.

## Results

**Fig 1:**
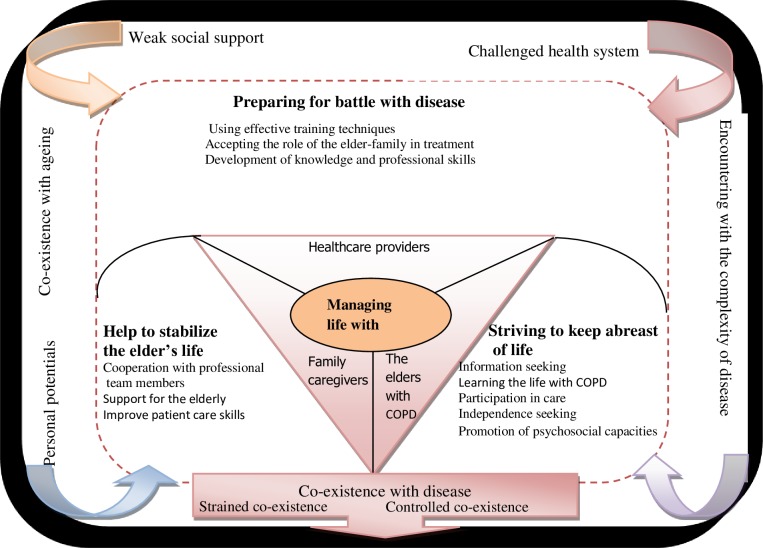
A schematic model of “Managing life with COPD” for affected elders.

The results showed that in encountering the complexity of the disease and in response to difficulties induced by COPD, the following three strategies were applied in “managing life with COPD” by the elderly persons with COPD, their family caregivers, and professional team members, respectively (Table 1): “striving to keep abreast of life,” “preparing for battle with disease,” and “helping to stabilize the elder’s life.” The outcome of these strategies was “co-existence with disease” (Fig 1). The potential of “managing life with COPD” was influenced by mediating factors, namely, “co-existence with ageing,” “personal potential,” “a challenged health system,” and “weak social support.” In this process, the mediator of “personal potential” led to some people reaching “controlled co-existence” and some elders, “strained co-existence,” because of barriers such as “a challenged health system” and “weak social support.”

The theory of “Managing life with COPD”: The empowerment process for elderly patients with COPD suggests that COPD and aging cause progressive disability among this subgroup. They have to learn to live with the disease and do so with three main strategies, namely, “striving to keep abreast of life,” “preparing for battle with disease,” and “helping to stabilize the elder’s life.” In relation to the core variable, a participant uses a metaphor to describe himself as a mechanic who can learn about the correct positions of bolts and nuts over time:

“*It is like a mechanic who learns about bolts and nuts over time*, *because you have to manage your life with these drugs*.” (Participant 4, a 60-year-old man)

### Striving to keep abreast of life

Sometimes, elderly patients with COPD might face significant losses as a result of developing COPD-induced disability and other conditions that are common among the elderly. Facing loss of capacities and deterioration due to ageing can also be very demanding. All these hardships can evoke suffering for the elderly and necessitate a response against a threat to oneself or one’s being or existence. This requires them to seek a remedy. So at first, all resources are mobilized, so elderly patients can learn how to live with the disease. The doctor will attempt to prescribe medication and provide general information, whereas the nurse, physiotherapist, and the dietician can help the elderly “learn to live with the disease.” To facilitate “participation in the process of care,” the elderly patients must trust healthcare providers. Among these, physicians are almost the most trusted. The chief nurse said:

“*The patient arbitrarily agrees with the physician and always the physician’s side and always accepts what he/she says*, *compared to nurses*. *It does not matter that nurses are also literate*.” (Participant 17, a nurse)

Striving to keep abreast of life consisted of five sub-categories, namely, information seeking, ‘learning to lead a life with COPD’, participation in care, independence seeking, and promoting socio-psychological capacities (Fig 1).

#### Information seeking

Along with the efforts of healthcare providers, elderly persons or their families seek information to meet their needs. They sought the required information from various sources, including “professional and non-professional resources,” and “the mass media,” which includes television, satellite, and the Internet. Illiterate participants used the radio as an information source. Some participants obtained the information from the nurses. However, in many cases, the participants used easily accessible non-professionals and peers who, in some cases, can give misleading information to the elderly patient. In this regard, one participant said:

“*When I spoke with friends and relatives and told them that I had this problem*, *they taught me a lot and have increased my knowledge*.” (Participant 1, a 70-year-old man)

An elderly smoker said:

“*My friends say that I should not suddenly quit smoking*, *or it will be problematic*.” (Participant 2, a 71-year-old man)

They also used “their experiences” in “continuing with normal life.” In some cases, due to the long history of the disease, the elderly persons had gained valuable experiences in self-management. In response to questions such as, “How did you get the information that you required?,” participants responded, “I know!” This indicated that the information was obtained through personal experiences. Another elderly man believed that he could prevent flu with self-care and without vaccination, and said:

“*I was careful not to catch a cold or not to do too much work*, *and so on*, *or I smoked less … Well*, I do not leave home, to avoid catching a cold; *I am always at home*. I have taken care of myself. *I have never had a vaccination and I never caught a cold*.” (Participant 11, a 73-year-old man)

#### Participation in care

Participants started participating in the process of care once they trusted healthcare providers. Despite the fact that knowledge about one’s condition is necessary, so as to engage in self-care, in most cases, participants did not have sufficient knowledge about the disease. Moreover, teams of healthcare providers believed that, in order to control the disease, knowledge alone is not sufficient and that it is necessary for elderly persons to apply their self-care knowledge. These teams were trying to help the elderly people manage their lives. The elderly people need to use drugs, oxygen, and Bi-PAP at home. However, they can only meet their needs:

“*If they feel breathless*, *they could use the devices for initial treatment at home*. *… When experiencing breathlessness*, *I use oxygen*, *spray myself*.” (Participant 7, an 80-year-old woman)

However, in some cases, the participants did not follow the recommended lifestyle due to some other reasons. An instance of this was the inability by all participants to quit smoking, due to psychological dependence and other deterrent factors. These factors included lack of knowledge, impatience, disability, dementia, drug shortages in the market, and financial problems. There is also a common form of self-treatment by elderly patients that is detrimental to disease control:

“*Self-treatment is very common among many of their patients*. *For example*, *there was a patient who used dexamethasone several times and took the corticosteroid*. *He was later faced with a horrible situation*.” (Participant 15, a nurse)

“Frequent hospitalization” and the inability to control disease shows lack of self-management ability.

“*I just started taking 50 mm of corticosteroids*, *as well as the antibiotics that I got there*, *and then I decreased the corticosteroid dose*, *based on my recovery*. *I was successful twice*, *but not the third time*.” (Participant 4, a 60-year-old man)

#### Independence seeking

Another subset extracted was, “Independence seeking,” which includes the subcategories, “efforts to reduce dependency” and “insistence on abilities.” In many cases, dependency may make the family relinquish all responsibilities of treatment and care of the elderly patient. However, less power largely induced aging and disease. Living alone was also stated as a problem. Despite receiving substantial support from her children, an elderly woman said:

“*When one of my children lived with me, it was easier and more comfortable.*” (Participant 5, a 77-year-old woman)

One of the problems mentioned by some older patients who lived alone was “dependency upon discharge from hospital”:

“*We tell them to leave because it may cause some kind of dependency*. *They know that you will come every day to remind them to use the spray*, *but they would not have such a thing at home*.” (Participant 17, a nurse)

In other instances, the elderly patients simply wanted to live alone and felt more comfortable doing so; an elderly woman said:

“*I feel comfortable in my own home*. *If I go to my son’s home*, *I cannot stay for more than a day*. *I go home the next day*.” (Participant 6, an 80-year-old woman)

“*I do all my own work*. *I do not need other people and I am not dependent*. *I am not bedridden and do not need my wife or kids to do things for me because of my disease*.” (Participant 1, a 70-year-old man)

Participants believed that independence was possible, as shown by “an insistence on their abilities.”

“*When a man knows that his body can take care of itself*, *then he should be careful not to eat or use the things that are harmful for his body*. *For example*, *I now know that smoking is bad for me; for the sake of my condition*, *I hate it and will not smoke anymore*.” (Participant 1, a 70-year-old man)

They made the decisions based on their beliefs and knowledge regarding their own care and treatment. In some instances, the decisions were useful and reasonable. In other instances, they attempted to make decisions that were contrary to their empowerment, that resulted in poor disease control:

“*This time*, *the doctor told me to buy the device (BiPAP) for home use*, *but I am not in a bad mood*. *I think that the device is for those who are in a very bad mood*.” (Participant 1, a 70-year-old man)

#### Promoting socio-psychological capacity

Additionally, participants tried to improve their socio-psychological capacity through “communication,” “having fun,” and “adaptation,” to improve their psychosocial capacities.

“*That is right; the disease has been bothering her for months*, *but I will do my best to make her well*, *to live with her happily like in the past*. *Well*, *we used to go to the park every morning before November; we did it to boost my mom’s morale…*.” (Participant 6, a family caregiver)

### Preparing for battle with disease

**Fig 2:**
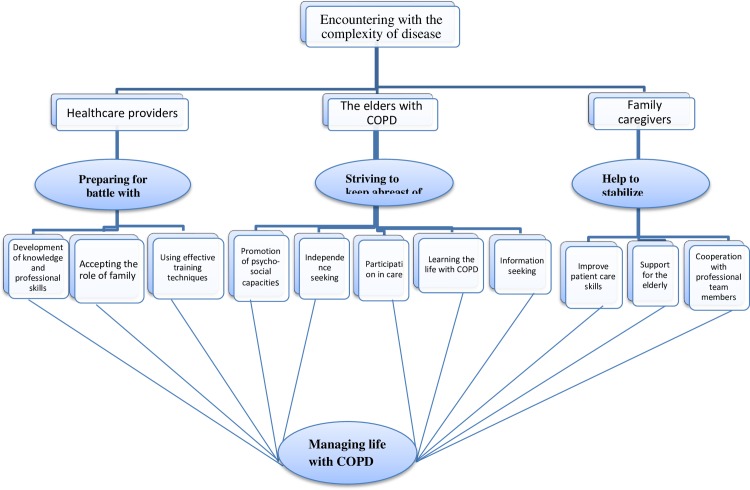
Managing life with COPD.

Preparing for battle with disease consisted of three sub-categories, namely, “using effective training techniques,” “accepting the role of the elder family member in treatment,” and “development of knowledge and professional skills” (Fig 2).

#### Using effective training techniques

In the empowerment process, healthcare providers’ efforts to involve elderly people in the treatment entailed use of strategies such as “giving information,” “striving to reduce dependency,” and “participation in disease management.” This ensures that healthcare providers, especially physicians, employ different ways to inform ill elderly patients about the disease, treatment, diagnosis, physiotherapy and pulmonary rehabilitation, oxygen therapy, how to use Bi-PAP, and self-care. In some cases, information was provided at the time of diagnosis. In this regard, one of the participants said:

“*I asked about what I can eat; should I follow a diet for my disease*? *The doctor told me ‘no*, *you can eat everything*, *you do not need any diet*.*’*” (Participant 5, a 77-year-old woman)

The healthcare providers know that, during training, they are faced with an aging audience that may find the teaching material difficult. Use of “techniques for addressing the aging audience” is one of the ways in which healthcare providers can provide effective training. In this regard, the physiotherapist said:

“… *Every day we go through the exercises that we taught them the day before*, *and we ask their family members whether the exercises were completed or not; we teach the elderly patient how to do these exercises again and try to check if they do them correctly*.” (Participant 18, a physiotherapist)

However, the healthcare providers know that the various training techniques taught should be based on the status of the elderly person. Even so, in some cases, unlike the professional team’s claim, client status was not considered. In this regard, one of the elders said:

“*A physiotherapist once came to our room and I was taking a lot of medication and if ‘I felt weak*. *He/she told me how to breathe*, *but I really was not in a good mood*. *The physiotherapist does not understand your situation*.” (Participant 4, a 60-year-old man)

The healthcare providers used different ways to ensure that the elderly were learning. “The nurse and physiotherapist control the patient’s learning” is a way in which team members ensure that elderly patients with COPD learn. The healthcare providers used educational aids for improved understanding of the material being taught. So, the format and language used in the instructional pamphlets used in field observations were easy to follow; these detailed how to use the spray and home-oxygen therapy. Moreover, the learning material was comprehensive, teaching both elderly persons and their families; the presence of families is necessary during all the educational processes:

“*Initially*, *during the first 15–20 minutes*, *we only check what they learned the previous time or whether they learned anything or no*, *and also examine if they perform activities correctly*. *Then*, *we ask questions to see how much they are learning*. *After ascertaining about their learning*, *we decide what to do for their physical therapy* … *because of their conscious fluctuations*, *one day they are sensible and the other day they are confused; this is why we have to teach their families*, *too*.” (Participant 18, a physiotherapist)

In rare cases, medications were provided with training. In this regard, an elderly patient said:

“*The doctor gave me a pill and told me to put it in the rice-cooker and switch power on*, *then take a sheet on it and sniff the steam*.” (Participant 7, an 80-year-old woman)

#### Accepting the role of the elder family member in treatment

Other than information by healthcare providers, who are trying to reduce dependency, participation of the elderly and their families is also required in treatment. However, the elderly and even family members do not play a role in treatment decisions. This served as a reminder to ask questions about the participation of the elderly or their families in treatment decisions. In this regard, one participant said:

“*We are providing training on tobacco cessation*, *no exposure to very cold or hot environments*, *no disposal of stimulants*, *[and] drug use*, *and they cooperate with us*.” (Participant 16, pulmonologist)

#### Development of knowledge and professional skills

The strengthening of ethical and professional obligations resulted in some people receiving treatment, despite their lack of power, and working hard to educate and empower seniors for their own benefit and the benefit of elderly sacrifice.

“*There are two physiotherapists in the hospital*, *but they are responsible for the ‘whole hospital*… *like’ or ‘there is staff shortage'*, *but they try to work with all patients*.*”* (Participant 15, head nurse)

### Help to stabilize the elder’s life

In the fight against COPD, the family and the elderly cooperate to overcome the disease, as if the disease has become the most important concern of the family and has affected family members’ lives. The family makes various efforts towards the elderly person’s well-being, such as cooperation with professional team members, support provision to the elderly, and improved patient care skills. Family caregivers of elderly people tried to stabilize the lives of the elderly through “cooperation with the team,” “support for the elderly,” and “improved patient care skills” (Fig 2).

#### Cooperation with professional team members

Given the importance of families in the process of empowerment, participants require families and professional teams to work together. Family members participating in the process of empowerment to ensure the effectiveness of the professional team were aware of this duty to cooperate:

“*Elders who have good family support get better answers than those who live alone; not a hundred percent*, *but it is less likely*. *In addition*, *an elderly who may not see or hear well does not have a good relationship with the outside world*.” (Participant 16, pulmonologist)

#### Support for the elderly

Faced with a complex disease in old age, elderly people were more dependent than at any other time. In addition to helping older family members with the process of striving in the course of life, another important approach was “helping to stabilize whose life,” so as to improve the lives of the older people receiving help. In order to enhance elderly persons’ psychosocial capacity while they are striving in the course of life and searching for information, families seek to enhance the elders’ psychosocial capacity. In the struggle with COPD, elderly people and their families have to cope with and overcome the disease, due to the disease being the most important concern of the family and affecting the lives of family members. Various attempts are made to ensure the well-being of elderly patients, to provide them with physical, emotional, and financial support, and to ensure that their living conditions are comfortable. Taking care of elderly family members brings satisfaction to family members and the elderly are also happy to pay more. Although dependent elderly family members have delegated many responsibilities to the family members, most of them had no complaints in this regard and made the utmost efforts to be expedient. They exerted efforts in caring for elderly persons, were satisfied, and considered it a blessing. They also seek to ensure the welfare of elderly persons by providing them with invaluable physical, emotional, and financial support.

*“People who receive good family support and care from their children get better answers are better off than those who live alone; not a hundred percent*, *but 'it' is less likely*. *In addition*, *an elderly who may not see or hear well is not in a good relationship with the outside world*.” (Participant 16, pulmonologist)

#### Improved patient care skills

Cooperation with families in treatment and their role as providers of family support necessitate that family caregivers improve their skills in patient care. Therefore, it is also important that through either self-study or practical application, family members obtain the knowledge and skills required to improve patient care to elderly people living with the disease to the elderly living with the disease too.

“*When I saw my mother’s disease progress day by day*, *I followed up with the doctor*. *I know a lot about my mother’s disease*, *about drugs*, *respiratory aid devices*. *As the saying goes*, *I am an ‘expert*.”(Participant 12, a family caregiver)

## Discussion

The theory of “Managing life with COPD” explains elders’ struggle with self-regulation and the strategies that they use to manage their disease. A difficult disease shapes their lives and governs their capacity to engage in activities of living. This is consistent with the findings of other researchers, who found that 'elderly persons' experiences with COPD warrants the latter’s definition as a “difficult disease elderly with COPD’s experience” [5, 14, 15]. Striving for self-regulation dictated every aspect of the elderly persons’ lives, including self- care, social life, autonomy, and family. Similar to the other studies, participants in this study spoke about their attempts to control their symptoms [16]. The study showed that older people’s daily lives were strongly influenced by the impact of the disease and ageing. They used various strategies to control their symptoms. The importance of “knowing what works” [14] for them personally was central to how they managed their disease. The likelihood of elderly persons with COPD maintaining and sustaining “autonomy” is greater if they know what to do, when, and how. However, the results showed that many elderly people did not know their correct diagnosis and the name and nature of their illness. This is because professional team members, especially physicians, used common words to enable better understanding by the elderly patient, leading to many elderly people thinking that they have asthma, instead of COPD. This result is harmonious with the results of Boots’ study, which showed that patients did not know their correct diagnosis [17].

The participants described knowledge acquisition as essential for their empowerment, a finding supported by previous researchers [18, 19]. They sought information from literature, the Internet, mass media programs, and peers, and by talking to healthcare providers. Patients have previously described seeking information outside healthcare encounters, so as to acquire an understanding of the disease [19]. Previous findings have underlined the need for healthcare providers to support patients by providing relevant knowledge that is important to them [10, 20].

Besides family members’ support to older people, health professionals were also reported as important resources in the elder's ability to cope with COPD. The characteristics of healthcare providers that elderly people perceived as empowering included delivering expert information and applying different educational strategies to elderly patients and their families. From the healthcare professionals’ perspective, they empowered elderly people and their family members by taking time to talk to them, answering questions, and offering helpful information. Furthermore, according to previous research, it is important that family members get the same information as the elder, to facilitate their understanding of the elder’s situation [19]. Healthcare providers have a responsibility to help elders with COPD and their families learn how to better control the former’s disease, as opposed to letting it control them. Empowerment programs that focus on teaching the elderly with COPD (e.g., the inhaler technique, working with the oxygen machine, and Bi-PAP) strategies to manage breathlessness (e.g., rehabilitation) and how to make lifestyle changes (e.g., exercising) show encouraging results. This result is consistent with those of other studies [21, 22].

Presumably, the elder’s self-regulation is a result of family support and the professional team’s efforts, which help motivate older people with COPD to exercise autonomy regarding their healthcare problems. These actions aim at a variety of issues, namely, coping (both problem-focused and from an emotion perspective) with and living with the disease, taking medication on time, managing medication side effects, and keeping oneself safe (including preventing or controlling the disease). This finding is harmonious with that in the study conducted by Sohanpal [23]. In this study, trust in the medical team, especially the physician, was found to be important to older people with COPD and their family members. This also had an impact on “adherence to treatment regimens” and the perceived quality of care. In this regard, although some elderly persons had succeeded in quitting smoking spontaneously on the advice of a doctor, others had made repeated unsuccessful attempts to quit without the benefit of a smoking cessation service. There is some evidence to suggest that, in general, older smokers are less likely to make a cessation attempt than are younger smokers; once they do, they are likely to be able to quit for three months or more [24, 25].

The elderly people with COPD in this study were striving for health related quality of life(HRQOL) by trying to control different types of symptoms in different ways of living with COPD, such as getting involved in treatment, maintaining independence, collaboration with the professional team, participating “in the process of care,” and the promotion of psychosocial capacity. The importance of maintaining independence was reiterated throughout the interviews. This finding is consistent with that in the study reported by Fonseca (2010) in relation to the lives of the elderly, with emphasis on the elderly person exercising his/her functional capacity by engaging in daily activities [26]. This seemed to be true for the participants in this study, as they struggled with many bodily problems. Their ill and ageing bodies certainly set limits for their lives. This result is the same as that in Hallberg’s study, wherein women with vertebral fractures were striving to maintain their independence by trying to manage different types of symptoms in different ways [27].

The nature of COPD means that control is forever in flux; for example, an acute exacerbation could temporarily shift the person from having “self-efficacy” to “dependence on care.”

The results of this study showed that demographic, familial, and social factors have important roles in the process of empowering elderly persons with COPD. These factors occasionally facilitate the empowerment care process and sometimes make it difficult [28]. By understanding these factors, healthcare providers can support elderly persons with COPD in managing to maintain independence in self-care and controlled co-existence with disease. Reducing the number of admissions, referral of elders with COPD to medical centers and clinics, and the financial burden of the disease, as well as increasing life satisfaction can be a consequence of empowerment interventions [29]. The results of this study can also be used in the formulation of policy and setting the standards of care in our country, as they illuminate important concepts and provide basic knowledge of the process of empowerment among elderly persons with COPD.

## Conclusions

“Self-regulation” enables the elder to feel in control and live optimally. This is a fragile balance, however, and the unpredictability of COPD can tip the elder into “self-efficacy.”

## Relevance to clinical practice

Nurses and other healthcare staff need knowledge and understanding of the meaning of empowerment in care, in relation to the ability of elders with COPD to meet their needs. Understanding the experiences of the empowerment process of elderly persons with COPD can help health professionals provide more focused elderly care.

## Supporting information

S1 Fig A schematic model of “Managing life with COPD” for affected elders. (TIF)

S2 Fig Managing life with COPD. (TIF)
