# Supplementary figures and images for: The empowerment of elderly patients with chronic obstructive pulmonary disease: Managing life with the disease
Source: PLoS One. 2017 Apr 3;12(4):e0174028. doi: 10.1371/journal.pone.0174028 (PMC5378379; doi:10.1371/journal.pone.0174028)

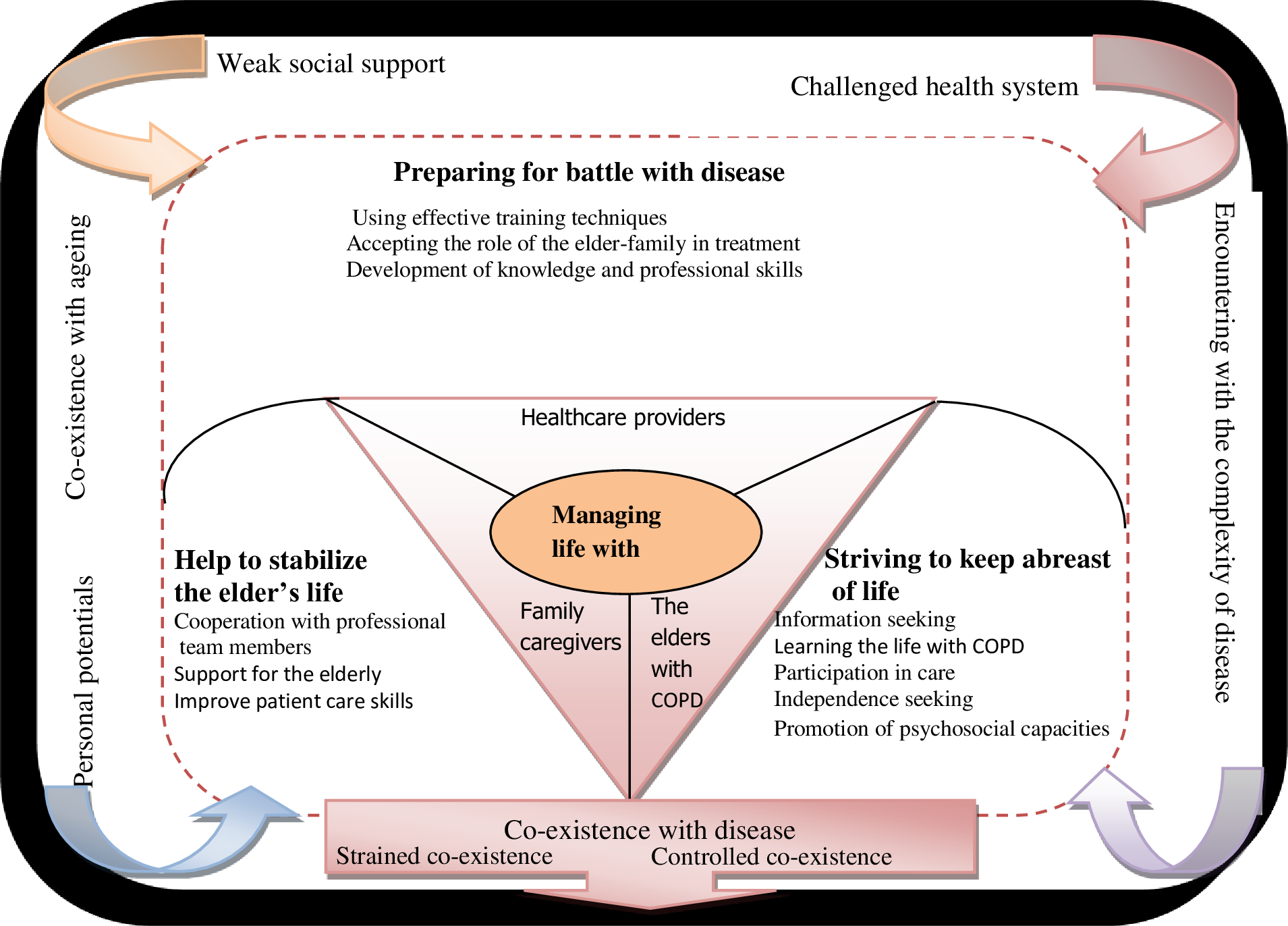

Supplement: S1 Fig — (TIF) [file pone.0174028.s001.tif]

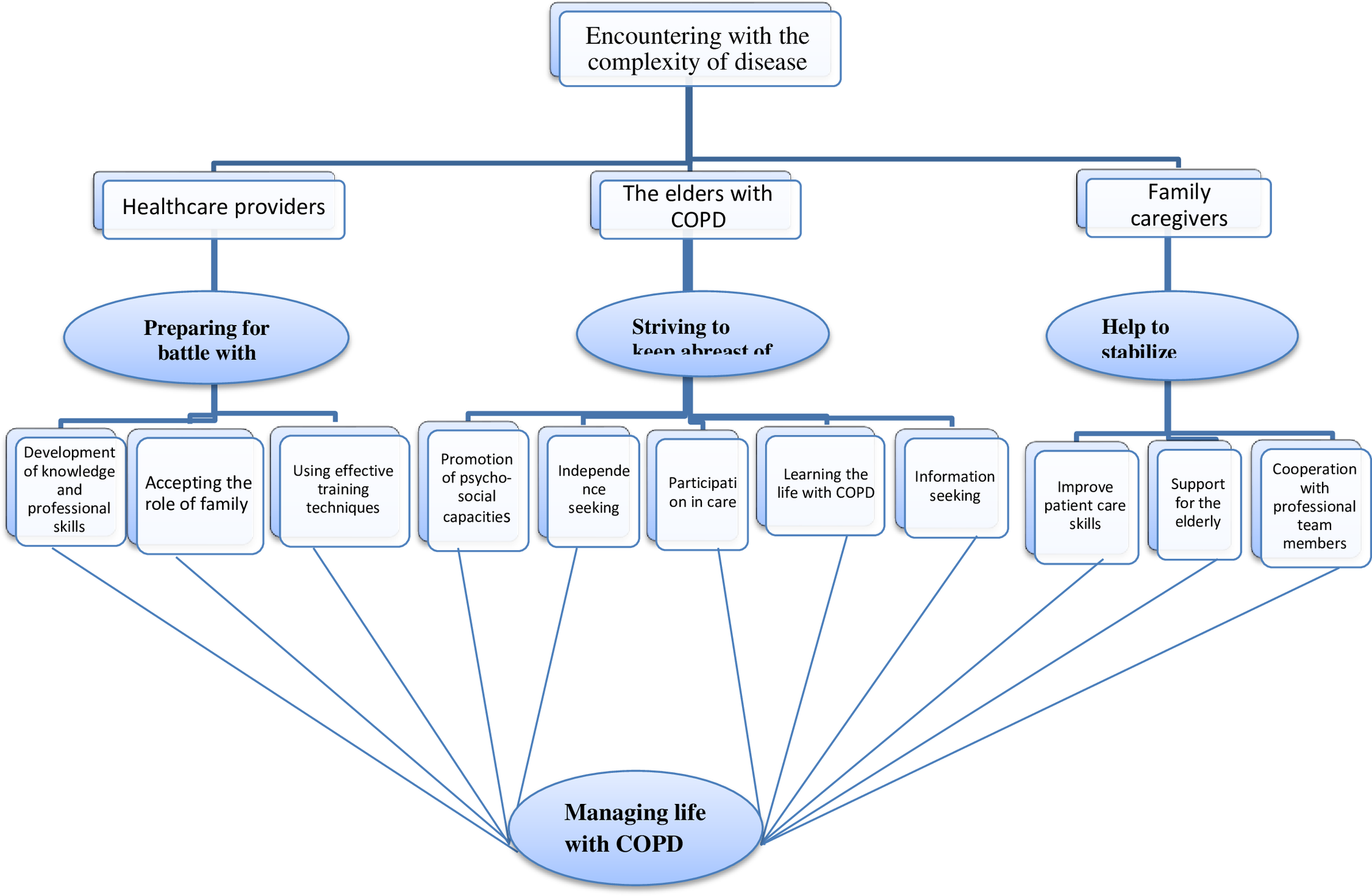

Supplement: S2 Fig — (TIF) [file pone.0174028.s002.tif]
